# Analysis of phase shift between pulse oscillations of macro- and microvascular cerebral blood flow in patients with traumatic brain injury

**DOI:** 10.1007/s00701-024-06209-5

**Published:** 2024-08-02

**Authors:** Magdalena Kasprowicz, Marta Hendler, Arkadiusz Ziółkowski, Nathalie Nasr, Marek Czosnyka

**Affiliations:** 1 Department of Biomedical Engineering, Faculty of Fundamental Problems of Technology, Wroclaw University of Science and Technology, Wroclaw, Poland; 2 Department of Neurology, Poitiers University Hospital, Poitiers, France; 3 Laboratoire de Neurosciences Expérimentales Et Cliniques, INSERM U-1084, University of Poitiers, Poitiers, France; 4 Division of Neurosurgery, Department of Clinical Neurosciences, Addenbrooke’s Hospital, University of Cambridge, Cambridge, UK; 5 Institute of Electronic Systems, Faculty of Electronics and Information Technology, Warsaw University of Technology, Warsaw, Poland

**Keywords:** Cerebral blood flow, Transcranial Doppler, Pulse oscillations, Laser Doppler flowmetry, Traumatic brain injury, Intracranial pressure

## Abstract

**Purpose:**

After a traumatic brain injury (TBI), monitoring of both macrovascular and microvascular blood circulation can potentially yield a better understanding of pathophysiology of potential secondary brain lesions. We investigated the changes in phase shift (PS) between cardiac-induced oscillations of cerebral blood flow (CBF) measured at macro (ultrasound Doppler) and microvascular (laser Doppler) level. Further we assessed the impact of intracranial pressure (ICP) on PS in TBI patients. A secondary aim was to compare PS to TCD-derived cerebral arterial time constant (τ), a parameter that reflects the circulatory transit time.

**Methods:**

TCD blood flow velocities (FV) in the middle cerebral artery, laser Doppler blood microcirculation flux (LDF), arterial blood pressure (ABP), and ICP were monitored in 29 consecutive patients with TBI. Eight patients were excluded because of poor-quality signals. For the remaining 21 patients (median age = 23 (Q1: 20–Q3: 33); men:16,) data were retrospectively analysed. PS between the fundamental harmonics of FV and LDF signals was determined using spectral analysis. τ was estimated as a product of cerebrovascular resistance and compliance, based on the mathematical transformation of FV and ABP, ICP pulse waveforms.

**Results:**

PS was negative (median: −26 (Q1: −38–Q3: −15) degrees) indicating that pulse LDF at a heart rate frequency lagged behind TCD pulse. With rising mean ICP, PS became more negative (R = −0.51, p < 0.019) indicating that delay of LDF pulse increases. There was a significant correlation between PS and cerebrovascular time constant (R = −0.47, p = 0.03).

**Conclusions:**

Pulse divergence between FV and LDF became greater with elevated ICP, likely reflecting prolonged circulatory travel time.

**Supplementary Information:**

The online version contains supplementary material available at 10.1007/s00701-024-06209-5.

## Introduction

In the early phase following a traumatic brain injury (TBI), a sequence of pathological events initiates a secondary brain injury (SBI) cascade, affecting brain regions that were not initially impacted [23]. Various factors, including intracranial hypertension, ischemia, hypoxia, and secondary hemodynamic changes may contribute to the development of SBI [2]. Post-TBI disturbance to microcirculatory blood flow, potentially caused by astroglial swelling, compression of adjacent capillaries [3, 19, 27], or the formation of thrombi in the cerebral microcirculation [22], may significantly influence the progression of SBI. Hence, monitoring changes in microvascular circulation alongside traditional macrovascular cerebral blood flow velocity (FV) evaluation can potentially yield a better understanding of the mechanism of TBI.

In this study, we aimed to analyse changes of cerebral blood flow (CBF) in macrovascular and microvascular bed at the acute phase after TBI and determine how rising intracranial pressure (ICP) drives these changes. More precisily, we aimed to assess phase shift (PS_FV-LDF_) between cardiac-induced oscillations of CBF at macro- and microvascular level, using Transcranial Doppler (TCD) and laser Doppler flowmetry (LDF), respectively. It should be noted that TCD enables non-invasive, continuous long-term monitoring of cerebral blood flow velocity (FV) in basal cerebral arteries, not cerebral blood flow itself. However, as we focus on pulse waveforms of FV not on its absolute changes, the use of FV as a surrogate for CBF should not introduce significant error. Furthermore, recent research has demonstrated the utility of TCD in detecting reduced CBF in TBI patients [1, 16]. LDF provides continuous assessment of the relative changes in microcirculatory blood flow [7]. Despite the risk of artifacts [11], this technique yields significant added information on changes in cortical flux in TBI patients hospitalized in intensive care units [12].

Additionally, we aimed to investigate the association of PS_FV-LDF_ with the cerebral arterial time constant (τ), a novel TCD-derived parameter that theoretically reflects the transit time of blood volume within one cardiac cycle from the insonation point of the middle cerebral artery to the arterial-capillary border [6, 9]. Previous studies have shown that lower τ was predictive for delayed cerebral ischemia in patients after subarachnoid haemorrhage [25] and that its shortening occurs very early, even before observing  vasospasm [8]. Shortening of τ was also reported in many pathological scenarios including TBI [24]. Conversely, τ increased with increasing ICP [6].

In this study, we investigated the changes in CBF along the cerebrovascular bed in terms of phase shift between pulse seen in FV and LDF signals. Additionally, we explored how PS_FV- LDF_ is influenced by the rise in ICP. A secondary aim of the study was to compare the PS_FV-LDF_ to TCD-derived τ.

## Material and methods

### Patients

Consecutive patients admitted for moderate to severe head injury to the Neurosciences Critical Care Unit of Addenbrooke’s Cambridge University Hospital between 1992 and 1994 and monitored continuously using TCD and LDF were considered for inclusion. Using legacy data rather than prospective monitoring was necessary as LDF was utilized over relatively short period in 1990s on our NCCU. Problems related to invasiveness of LDF, lack of appropriate probe holders, introduction of invasive brain tissue oxygenation monitoring led to a decline in interest in cortical LDF. However, the uniqueness of the recorded signals prompted the authors to conduct a retrospective analysis of the database as a potential source of knowledge about the relationship between cerebral macro- and microcirculation.

All patients were sedated intubated and ventilated to a PCO_2_ of 3.5–4.0 kPa. The target value for the cerebral perfusion pressure in the treatment protocol was as follows: greater than 55 mm Hg in those patients with raised ICP using a constant infusion of dopamine (5–15 ug/kg/min). If this treatment failed, boluses of mannitol (200 ml of 20% over 20 min) were given and repeated as necessary. The management protocol was approved by Cambridge Health Authority’s local research ethics committee [12].

### Data collection and Signal Analysis

ICP was continuously monitored with parenchymal probes (Camino, San Diego CA, USA). An arterial line through the radial artery was placed for invasive continuous monitoring of ABP (20 G catheters, Arrow UK; transducers and monitors, S and W Denmark). Monitoring of cortical capillary flux was assessed with an LDF probe (MBF3D monitor and modified P3 probe; Moores Instruments, Axminster, UK) through the same burr hole as the ICP probe. LDF probes were positioned at a depth, where a maximal pulsatile cortical flux signal was achieved. MCA blood FV was measured with a 2 MHz pulsed, TCD probe (model PCDop 842; Scimed, Bristol, UK) fixed in position using a head holder.

**Fig. 1:**
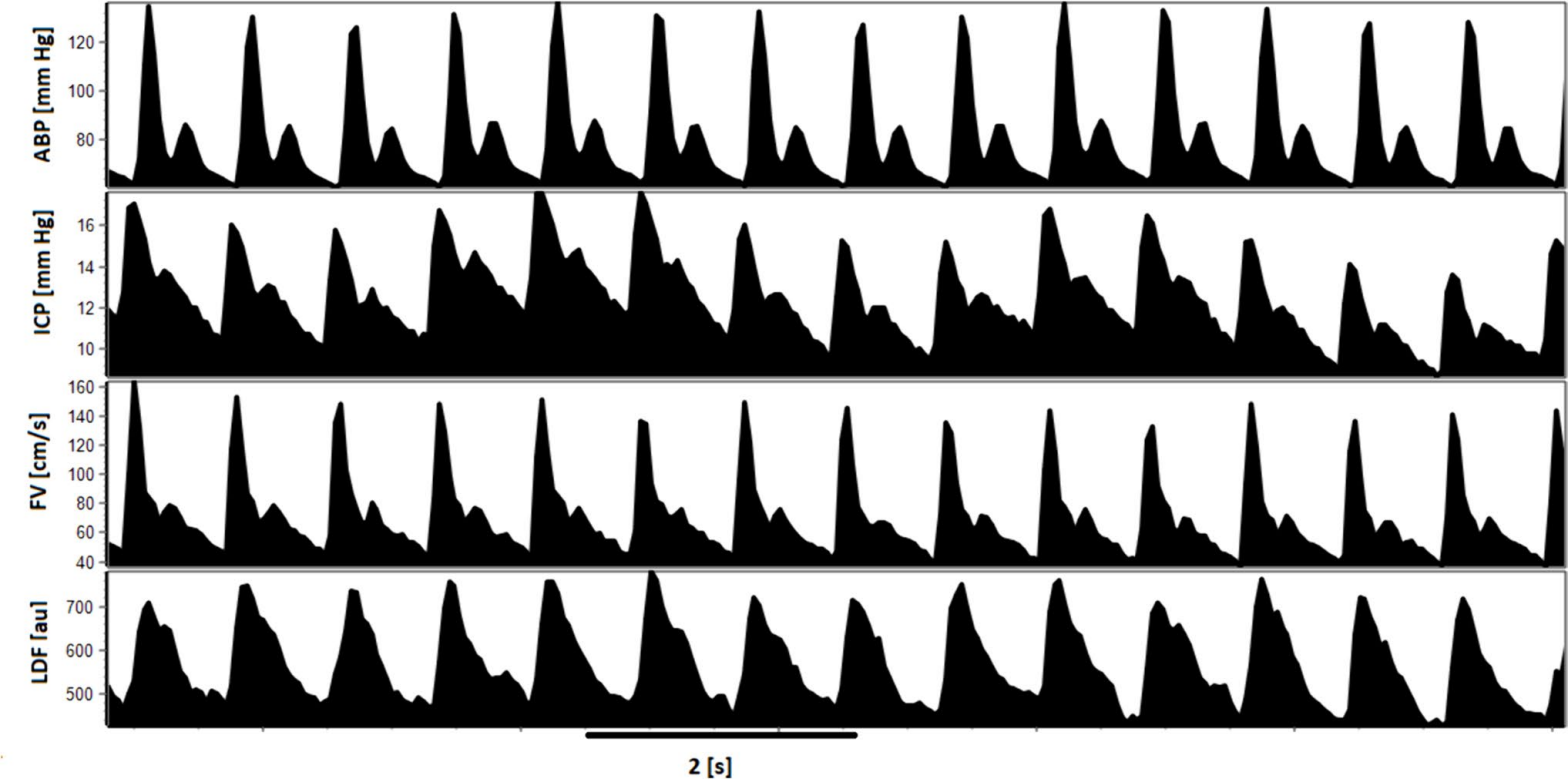
Pulse waveforms of recorded signals: ABP, ICP, FV and LDF. Abbreviations: ABP – arterial blood pressure [mm Hg], ICP – intracranial pressure [mm Hg], FV – cerebral blood flow velocity [cm/s]; LDF – laser Doppler flux signal [arbitrary units; au]

Signals of ABP, ICP, LDF, and FV were sampled at a frequency of 30 Hz, and calibrated in appropriate units (mm Hg, mm Hg, and cm/s, respectively), except for the LDF signal which was recorded in arbitrary units (au). Artifacts were removed after visual inspection. Good-quality signals (including visible cardiac-related pulses with distinguishable systolic and diastolic peaks) were further processed with ICM+ software (Cambridge Enterprise Ltd, Cambridge, UK). An example of recorded pulse waveforms is presented in Fig. 1.

### Assessment of phase shift between FV and LDF pulse waveforms using spectral analysis

**Fig. 2:**
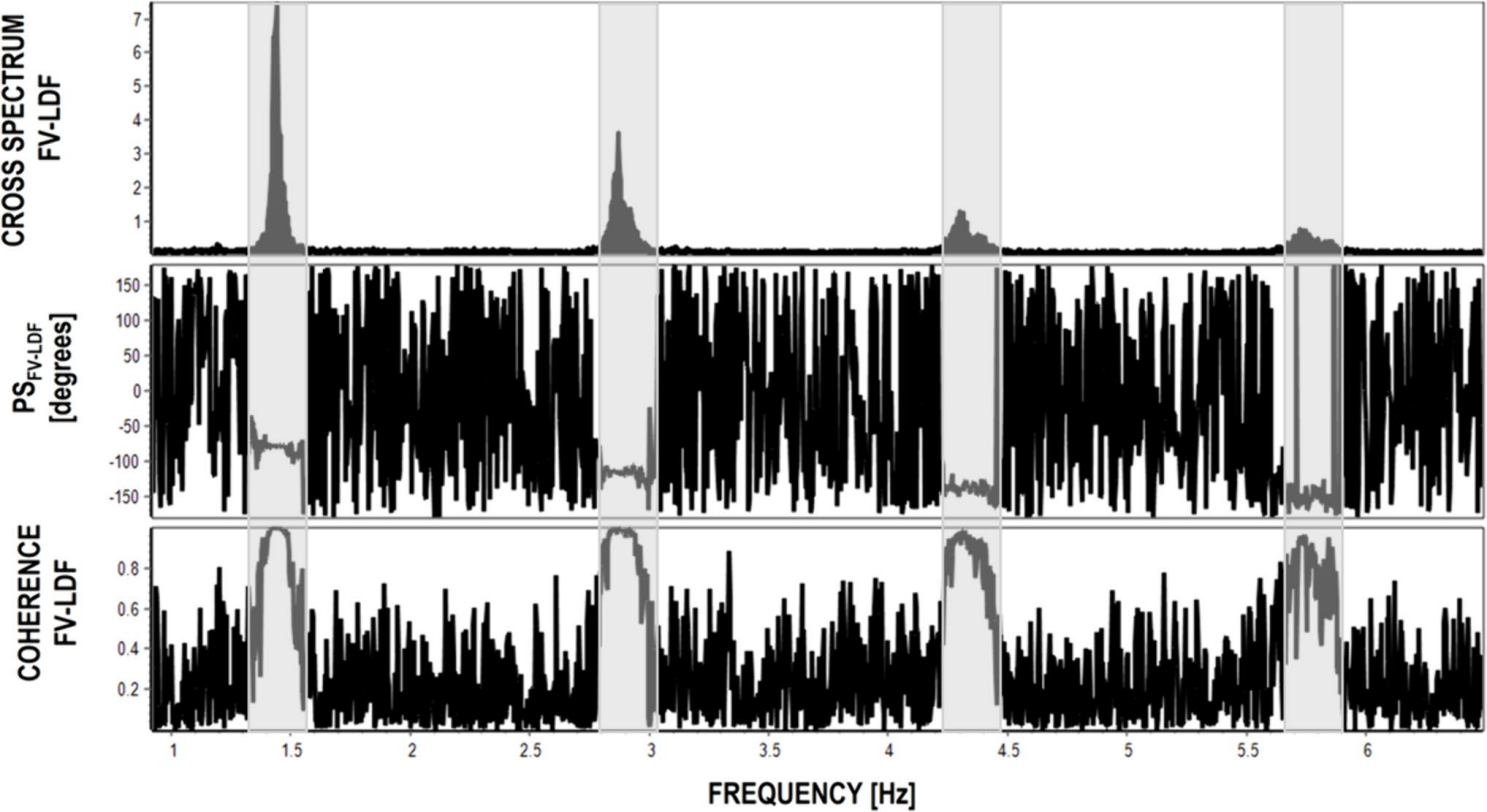
Cross-spectrum of FV and LDF signals (*upper figure*). Phase shift between FV and LDF (*middle figure*). Coherence between FV and LDF (*bottom figure*). The cross-spectrum of FV and LDF signals shows peaks at frequencies equivalent to the heart rate and its higher harmonics (*areas shaded in grey*). Both phase shift and coherence are chaotic between peaks of cross-spectrum, but close to the peaks, they show consistent values. Abbreviations: FV – cerebral blood flow velocity [cm/s]; LDF – laser Doppler flux signal [arbitrary units; au], PS_FV-LDF_ – phase shift between FV and LDF pulse waveforms [degrees]

To determine the phase shift between pulse oscillations in FV and LDF signals (PS_FV-LDF_), the transfer function analysis method was used. This involved estimating cross spectra by transforming the time series of FV and LDF signals with discrete Fourier transformation to the frequency domain. The resulting complex-valued function provides information about the amplitude and phase shift at a frequency corresponding to heart rate and its higher harmonics, with the phase indicating the temporal lag between the input (FV) and output (LDF) signals. Coherence was also analyzed to determine the degree of linear coupling between these two signals at heart rate frequency (Fig. 2).

### The cerebral arterial time constant (τ)

Τ was estimated using a simplified cerebral circulation model developed by Czosnyka et al. [5] and defined as the product of the compliance of large cerebral arteries (C_a_) and the resistance of small regulatory arteries (CVR) during a cardiac cycle, according to the equation:

1 $$\tau ={\text{C}}_{\text{a}}\bullet \text{CVR}$$

Detailed explanations of the formulas employed to calculate τ have been described in earlier publications[6, 9, 26]. A brief description of both C_a_ and CVR calculation is provided in Supplementary material.

### Statistical analysis

Statistical analysis was performed using Statistica software (v13, Tibco, Palo Alto, CA, USA). Repeated measures obtained from the same patient were averaged to avoid the effects of multiple sampling. The normality of the data was assessed using the Shapiro-Wilk test. Because of limited observations and lack of normality distribution for most of the analyzed variables non-parametric tests were applied. Correlation between numerical data was assessed using Spearman’s rank test. Logistic regression was applied to investigate whether τ or PS_FV-LDF_ is associated with mild intracranial hypertension (defined as ICP ≥ 15 mm Hg) or severe intracranial hypertension (defined as ICP ≥ 20 mm Hg). We evaluated the model’s performance using constructed receiver operating characteristic (ROC) curves, with the area under the curve (AUC) serving as the evaluation metric. The level of significance was set at 0.05. Data were presented as medians (first–third quartile) unless otherwise indicated.

## Results

### Cohort characteristics

Twenty-nine patients were considered for inclusion. Eight patients were excluded due to the low quality of monitored signals (ICP:1 patient, FV: 2 patients, and LDF: 5 patients). Data from 21 patients (age = 23 (20–33), men: 16, Glasgow Coma Scale on admission (GCS) was of 5 (4–7)) in whom reliable and continuous ICP, ABP, LDF, and FV pulse signals had been recorded were further evaluated with a spectral analysis algorithm to determine the PS_FV- LDF_ and τ. A total of 33.5 h of data recorded in 62 monitoring sessions were analyzed.

### Relationship between ICP, CPP, PS_FV-LDF_ and τ

**Fig. 3:**
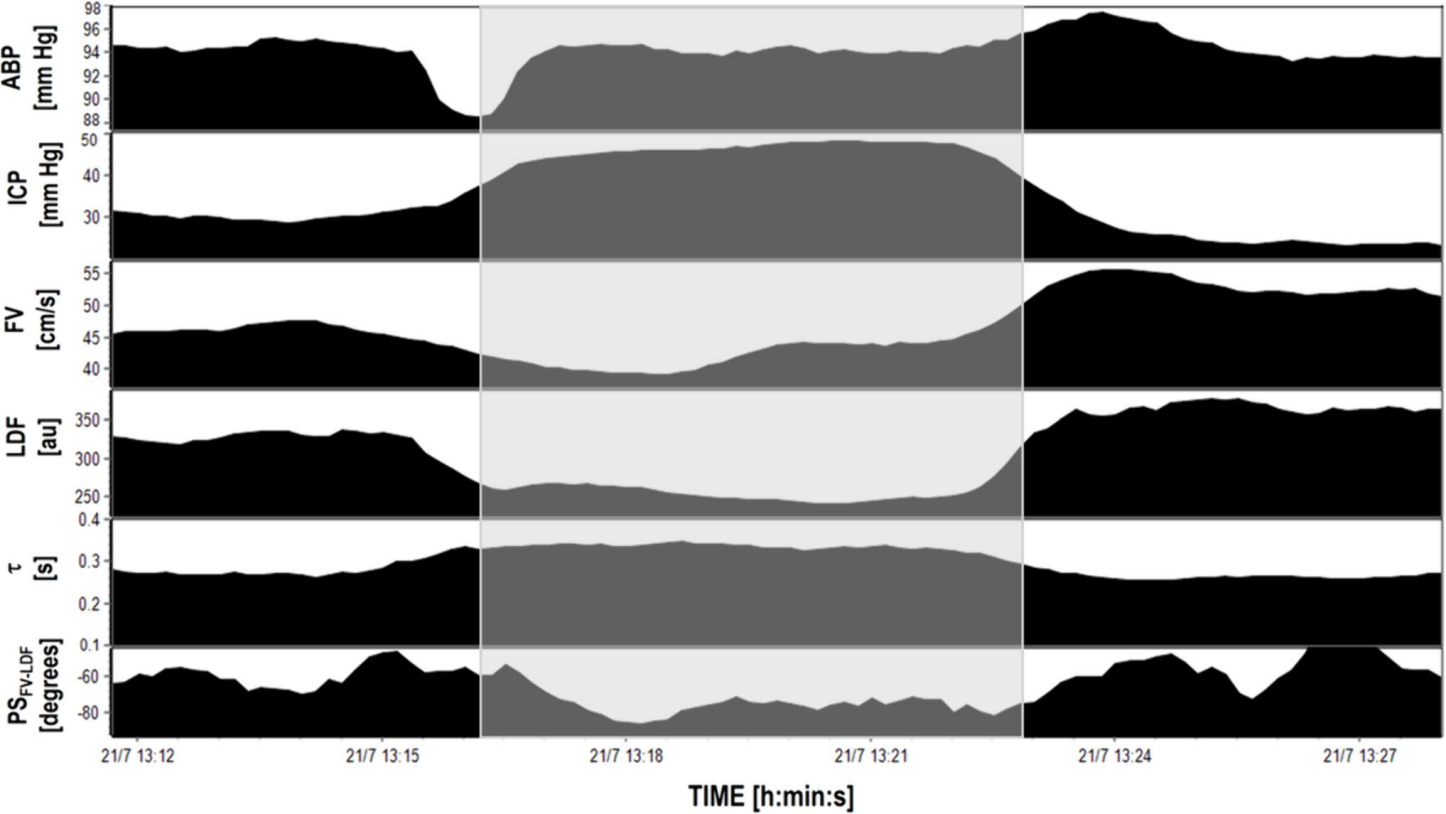
Changes in ABP, ICP, FV, LDF, τ, and PS_FV-LDF_ during an ICP plateau wave recorded in a single TBI patient. With rising ICP during the plateau wave (*area shaded in grey*) PS_FV-LDF_ became more negative while τ increased indicating a greater separation between FV and LDF pulses and a slower transition of blood volume throughout the cerebrovascular bed from macro to microvasculature, during one heart cycle, respectively. Abbreviations: ABP – arterial blood pressure [mm Hg], ICP – intracranial pressure [mm Hg], FV – cerebral blood flow velocity [cm/s]; LDF – laser Doppler flux signal [arbitrary units, au], τ – cerebral arterial time constant [s], PS_FV-LDF_ – phase shift between FV and LDF pulse waveforms [degrees]

Both PS_FV-LDF_ and τ presented similar changes during intervals of multimodal recordings. A recording during an ICP plateau wave along with accompanying changes in PS_FV- LDF_ and τ in single patient are presented in Fig. 3.

**Fig. 4:**
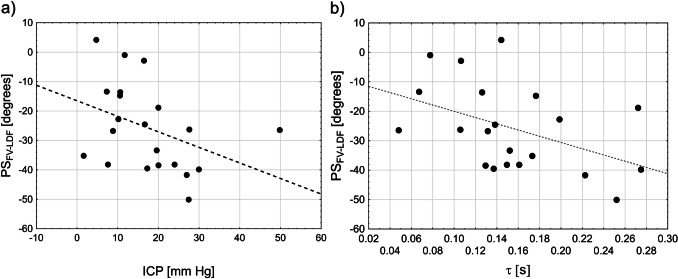
Relationship between phase shift (PS_FV-LDF_) between pulse waveforms of transcranial Doppler cerebral blood flow velocity (FV) and laser Doppler flux (LDF) and (a) cerebral arterial time constant (τ), R_Speraman_ = −0.47, p = 0.03 and (b) mean intracranial pressure (ICP), R_Spearman_ = −0.51, p < 0.019

**Fig. 5:**
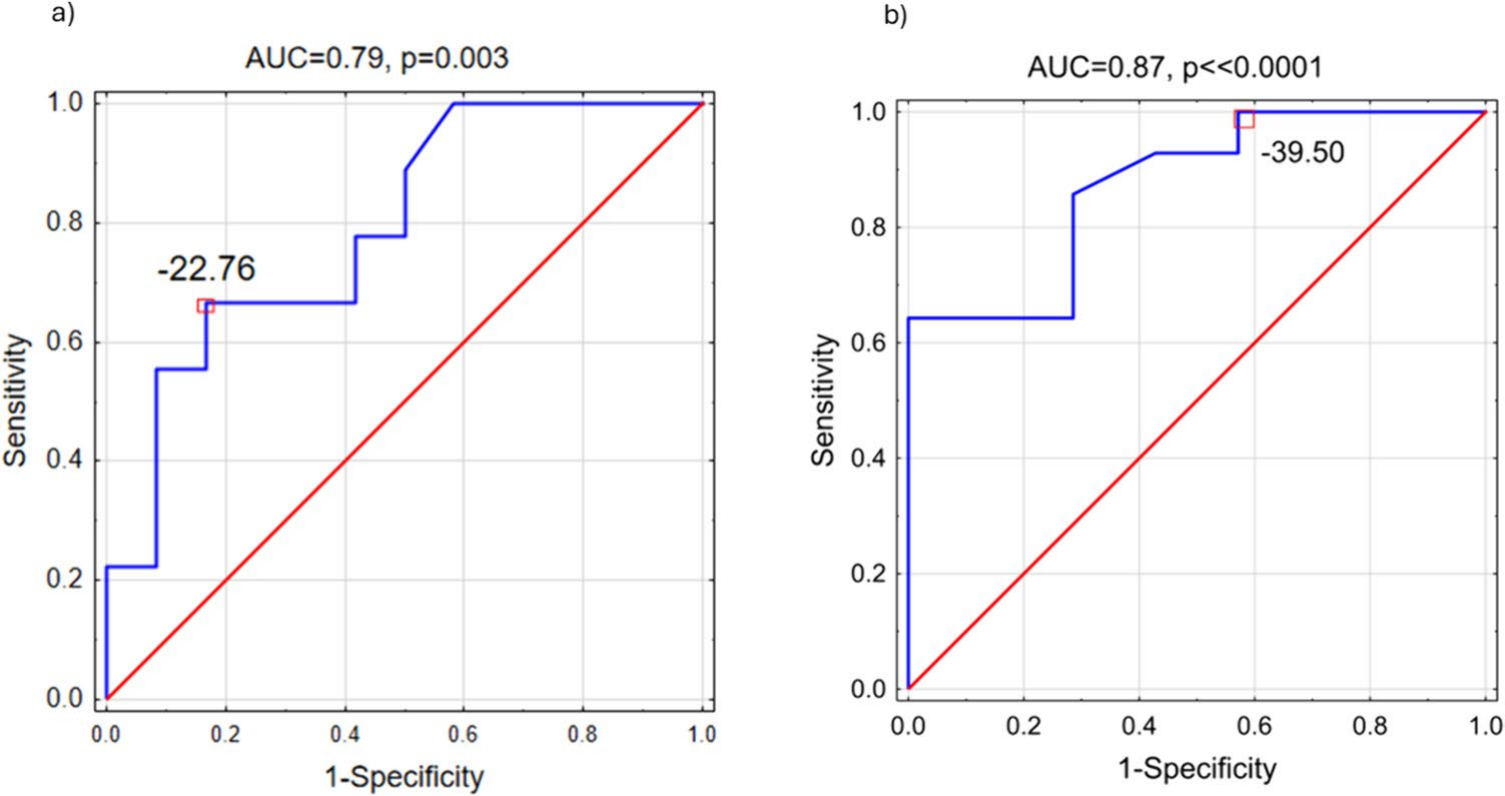
The Receiver Operating Characteristic (ROC) curve with area under the curve (AUC) for the association of phase shift between FV and LDF pulse waveforms (PS_FV-LDF_) with a) mild intracranial hypertension (ICP >  = 15 mm Hg) and b) severe intracranial hypertension (ICP >  = 20 mm Hg)

In group analysis, PS_FV-LDF_ was significantly correlated with τ (R_Speraman_ = −0.47, p = 0.03; Fig. 4 a). PS_FV-LDF_ was negative (Table 1) and became more negative in patients with higher mean ICP (R_Spearman_ = −0.51, p < 0.019; Fig. 4 b). Results of the logistic regression analysis demonstrated that PS_FV-LDF_ had statistically significant association with ICP elevation, both mild (OR = 0.92 [0.85–1.00], p = 0.05; AUC = 0.79, p = 0.003; Fig. 5a) and severe (OR = 0.86 [0.75–1.00], p = 0.035; AUC = 0.87, p <  < 0.0001; Fig. 5b). However, τ was neither associated with nor correlated with ICP elevation. Additionally, there was no significant relationship between cerebral perfusion pressure (CPP) and both τ and PS_FV-LDF_.

**Table 1:** Physiological values and calculated variables for 21 TBI patients

Parameter	Median (first–third quartile)
ICP [mm Hg]	17 (10 – 24)
ABP [mm Hg]	83 (78 – 93)
FV [cm/s]	57 (48 – 68)
LDF [au]	238 (192 – 353)
PS_FV-LDF_ [degrees]	−26 (-38 – −15)
Coh_FV-LDF_ [au]	0.55 (0.47 – 0.65)
τ [s]	0.144 (0.126 – 0176)

Abbreviations: ICP – intracranial pressure, ABP – arterial blood pressure [mm Hg], FV – cerebral blood flow velocity [cm/s]; LDF – laser Doppler flux signal [au, arbitrary units], PS_FV-LDF_ – phase shift between FV and LDF pulse waveforms [degrees], Coh_FV-LDF_ – coherence between FV and LDF pulse waveforms [au, arbitrary units], τ –cerebral arterial time constant [s]

## Discussion

Our results show that microvascular pulse oscillations assessed by LDF lag behind those of macrovascular oscillations assessed by TCD. This pulse divergence became greater with elevated ICP in patients with TBI. Our study also demonstrates that PS_FV-LDF_ has a good association with intracranial hypertension. Changes in PS_FV-LDF_ are correlated with changes in cerebral arterial time constant (τ) which is expected to reflect the transit time of blood volume along the cerebrovascular arterial bed within one heart cycle. Longer τ was associated with greater delay between macrovascular and microvascular pulse oscillations.

Following TBI, a complex series of pathological processes begin, often leading to secondary brain lesions. This secondary damage can be driven by several factors, such as increased ICP, ischemia, hypoxia, and changes in CBF dynamics both at macrovascular and microvascular level. Disruption of blood flow at microvascular level has been attributed to swelling of astrocytes, squeezing of nearby capillaries, or blood clots in the brain's small vessels[3, 19, 22, 27]. A diminished capacity of large brain's blood vessels to adapt to changes in perfusion pressure and a compromised ability to regulate blood flow at microvascular level are probably the main factors determining inadequate supply of cerebral blood flow to the brain.

Physiological monitoring of changes in ICP, brain tissue oxygen, and blood circulation in both the large blood vessels and the microcirculation are considered to be crucial for understanding the complex mechanisms of TBI and for designing optimal treatment strategies. However, despite substantial advances in multimodality physiological monitoring, management of TBI in the intensive care setting remains focused on physiological targets, including ICP, cerebral perfusion pressure, or cerebral autoregulation indices, even though the adverse effects linked to ICP elevation are unlikely to be uniform across patients and over time. In line with recent recommendations of the International Traumatic Brain Injury Research Initiative [13] future management of TBI patients, would benefit from moving toward patient-tailored care based on real-time multimodal monitoring to prevent secondary brain lesions. While ICP plays a significant role in TBI management and prognosis [18], other hemodynamic parameters are necessary to better understand both the impact of ICP on microvasculature and the secondary brain lesions that are partly independent of ICP. Recently, management of ICP has been broadened by incorporating additional physiological monitoring modalities to better individualize therapy. Monitoring of partial pressure of brain tissue oxygen (PbtO_2_) in addition to ICP was shown to reduce brain tissue hypoxia [14], with promising interventions aiming at optimizing oxygen delivery. Nonetheless, microcirculatory changes in cerebral blood flow in TBI and their role in the occurrence of cerebral hypoperfusion remain poorly understood, partially due to the lack of adequate investigational techniques often requiring invasive monitoring. The LDF technique we used, is invasive and is not a part of the routine hemodynamic monitoring in TBI at present. However recent advances in optical measuring techniques offer the possibility of replacing invasive LDF with other non-invasive modalities that are sensitive to microcirculation, such as Near Infra-Red Spectroscopy (NIRS) or diffuse correlation spectroscopy (DCS) [10, 20, 21]. Experimental studies in animals have demonstrated usefulness of DCS and NIRS techniques for estimating ICP changes [20, 21]. Considering that the delay between FV and LDF pulses (PS_FV-LDF_) seems to be a good predictor of intracranial hypertension, future work incorporating surrogate measurements of blood flow in the microcirculation may open possibilities for development of non-invasive method for detecting ICP rises. Still, additional research is needed to validate the effectiveness of these non-invasive techniques in accurately assessing CBF within the microcirculation. Time constant (τ), on the other hand, is a TCD-derived hemodynamic parameter that was found in our study to be correlated with PS_FV-LDF_. Time constant got longer with more negative PS_FV-LDF_ which means that prolongation of the transit time of CBF from macrovascular to microvascular bed is associated with greater dispersion between FV and LDF pulses. τ has been previously extensively studied in various clinical contexts [4, 6, 15, 24], demonstrating potential as a predictor for adverse outcomes like delayed cerebral ischemia [25] or vasospasm [8]. It could serve as an alternative to PS_FV-LDF_ in moderate TBI patients to assess cerebral changes in CBF in macro—and microvasculature and potentially the risk of occurrence of secondary brain ischemic lesions.

However, τ appears to be less sensitive than PS_FV-LDF_ to changes in mean ICP. One possible reason for this lack of correlation is that ICP levels in our TBI cohort were not high enough to induce changes in both C_a_ and CVR or/and an ongoing pathological process may have weakened the reactivity of both C_a_ and CVR to increased ICP. Our modelling approach might also underestimate changes in C_a_, as it assumes non-pulsatile venous outflow (please see Supplementary material), which may not apply in elevated ICP scenarios. Unlike the PS_FV-LDF_ estimation method, changes in microcirculation are not directly measured but approximated by the resistance of small cerebral vessels (CVR), determined as the ratio of CPP to mean flow velocity (FV) in basal arteries. Consequently, τ may not be as sensitive to changes in mean ICP as PS_FV-LDF_.

In this paper, we described results arising from invasive monitoring of cortical blood flow that potentially provide the missing links for a comprehensive approach to cortical perfusion assessment in TBI patients. Additionally, we proposed an alternative TCD-based methodology, for transit time estimation in TBI. To our knowledge, this is the first study in TBI on phase shift estimated from fast (cardiac-related) oscillations of the macro- and microvascular cerebral bed. The phase shift between slow changes at macro- and microvascular level has been previously observed in patients with carotid stenosis with the microvascular changes assessed with NIRS lagging behind macrovascular changes assessed with TCD during controlled breathing [17]. However, phase relationship between fast oscillations has been the blind part of cerebral hemodynamic assessment in clinical pathological contexts including TBI.

## Limitations

The limitations of his study are firstly, its retrospective design based on previous prospective data [11, 12, 28] and, secondly, the relatively small number of patients included. The second limitation was mainly due to the invasive nature of LDF which is not commonly used in current practice, leading to a relatively small amount of available data. Despite the development of signal analysis methods, there are no newer databases containing continuous recordings of FV and LDF pulse waveforms. Therefore, even though the database was registered in the 1990s and is relatively small, exploring the relationship between micro- and macrocirculation with its use seems justified. The limited use of LDF in current clinical practice may diminish the immediate clinical applicability of our findings. Nevertheless, we believe that presented results may contribute to the understanding of cerebrovascular dynamics in TBI and guide new research using non-invasive modalities that are sensitive to microcirculation, such as Near Infra-Red Spectroscopy (NIRS) or diffuse correlation spectroscopy (DCS).

Data on arterial pCO2, microdialysis and oxygen pressure were not available as the data were acquired at a time where the recommendations for monitoring were different. Equally important is to discuss the spatial limitation of LDF measurements, especially from a retrospective cohort where brain imaging data were not included.

Our results did not reveal a significant association between CPP and both τ and PS_FV-LDF_. However, the methods used for τ and PS_FV-LDF_ estimation may have interfered with the results, and a link might still exist. Moreover, transit time of cerebral blood flow can be influenced by factors beyond CPP, such as metabolic demand, blood gas levels, and neural activity, which might overshadow CPP's influence. Additionally, our small sample size may have hindered the detection of such a correlation.

## Conclusions

Phase shift between cardiac-induced oscillations of CBF measured at macro- and microvascular level was found to be negative in TBI and became more negative when ICP rises. This suggests that time lag between arterial pulse in big cerebral arteries and arterioles increases with intracranial hypertension. Also τ, a TCD-derived hemodynamic parameter was associated with phase shift. Hence, τ may be interpreted as a surrogate of phase shift in TBI patients to assess delay in cerebral blood flow in macro- and microvasculature.

## Supplementary Information

Below is the link to the electronic supplementary material.

Supplementary file1 (DOCX 66 KB)

## Data Availability

The data that support the findings of this study are available from the senior author, [MC], upon reasonable request.

## Abbreviations

**CBF**: Cerebral blood flow [ml/min]
**FV**: Cerebral blood flow velocity [cm/s]
**ICP**: Intracranial pressure [mm Hg]
**LDF**: Laser Doppler flowmetry
**PS**_**FV-LDF**_: Phase shift between pulse oscillations in FV and LDF signals [degrees]
**TCD**: Transcranial Doppler ultrasonography
**TBI**: Traumatic brain injury
**τ**: Cerebral arterial time constant [s]

## Code availability

The code in the form of a computational profile compatible with Intensive Care Monitor Plus (ICM +) software is available from the corresponding author, [MK], upon reasonable request.
