# Supplementary material for: Analysis of phase shift between pulse oscillations of macro- and microvascular cerebral blood flow in patients with traumatic brain injury
Source: Acta Neurochir (Wien). 2024 Aug 2;166(1):321. doi: 10.1007/s00701-024-06209-5 (PMC11297107; doi:10.1007/s00701-024-06209-5)
Supplement: Supplementary file 1 — Supplementary file1 (DOCX 66 KB) [file 701_2024_6209_MOESM1_ESM.docx]

**Supplementary material**

*Changes in cerebral arterial blood volume*:

The changes in cerebral blood volume (ΔCBV) during a cardiac cycle can be calculated by integrating the difference between pulsatile arterial inflow (CBF_a_) and venous outflow (CBF_v_) of cerebral blood:

$\Delta CBV \left( t \right)= \int_{t_{0}}^{t} \left( {CBF}_{a}\left( t \right)-{CBF}_{v}\left( t \right) \right)dx$ (1)

Where t_0_ denotes a beginning of a cardiac cycle and x denotes a integration variable.

As venous outflow is relatively low pulsatile (see Supplementary Figure 1), it can be expressed as the averaged arterial inflow (CBF_m_).


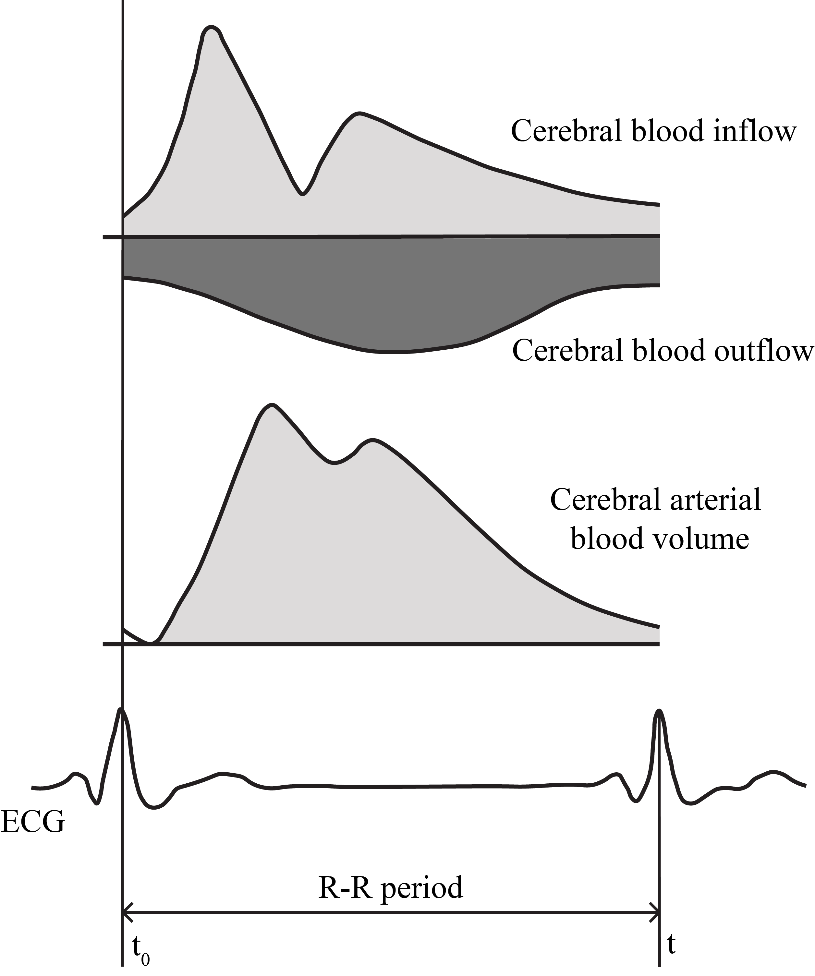


Supplementary Figure 1. Schematic visualization of pulsatile cerebral blood inflow, less pulsatile cerebral blood outflow and cerebral arterial blood volume over a single cardiac cycle ECG – electrocardiogram, t0 – beginning of a cardiac cycle, t – end of a cardiac cycle.

Taking into account the finite sampling frequency and assuming that the cross-sectional area of the insonated vessel is equal to S_a_, we can rewrite the previous equation as a discrete-time difference equation in terms of flow velocity (FV)

$\text{Δ}\text{C}_{a}\text{BV}=\sum_{\text{i=1}}^{m} (\text{FV}(i)-\text{FV}_{m})\text{Δt} [$cm] (2)

where m is the number of samples, Δt is the time interval between two subsequent samples, FV(i) are the samples of the cerebral blood flow velocity (FV), and FV_m_ is the moving average of FV from the window that includes several previous heart evolutions (a 6-second window was applied). ΔC_a_BV is normalized for further calculation (divided into the unknown S_a_ [cm^2^]), resulting in expression in [cm].

*Cerebrovascular resistance:*

Cerebrovascular resistance (CVR) is the resistance of small cerebral arteries and arterioles, defined as the ratio between mean cerebral perfusion pressure (CPP=ABP_m_-ICP_m_) cerebral blood flow approximated by TCD blood flow velocity (FV_m_).

$\text{CVR =}\frac{CPP}{{FV}_{m}} \left[ \frac{\text{mmHg}}{\text{cm/s}} \right]$ (3)

*Compliance of the cerebral arterial bed:*

Compliance of the cerebral arterial bed (Ca) is estimated as the ratio between the amplitude of ΔC_a_BV (Amp_C_a_BV) and the amplitude of ABP (Amp_ABP), as shown below:

$C_{a}\text{ =}\frac{Amp\_C_{a}\mathrm{BV}}{Amp\_ABP} \left[ \frac{cm}{mm Hg} \right]$ (3)

Where Amp_CaBV and Amp_ABP - the amplitudes of fundamental components (first harmonic) of CaBV and ABP pulse waveforms, respectively.
